# Supplementary material for: Considering socio-political framings when analyzing coastal climate change effects can prevent maldevelopment on small islands
Source: Nat Commun. 2021 Oct 7;12:5882. doi: 10.1038/s41467-021-26082-5 (PMC8497557; doi:10.1038/s41467-021-26082-5)
Supplement: Supplementary file 1 — Supplementary Information [file 41467_2021_26082_MOESM1_ESM.pdf]

Supplementary Information for the article:

Considering socio-political framings when analyzing coastal climate change effects can prevent maldevelopment on small islands

C. Gabriel David[1,*], Arne Hennig[2], Beate M. W. Ratter[2,3], Volker Roeber[4,5], Zahid[6], and Torsten Schlurmann[1]

[1]Ludwig-Franzius-Institute for Hydraulics, Estuarine and Coastal Engineering, Leibniz Universität Hannover, Nienburger Straße 4, 30167 Hanover, Germany
[2]Institute for Geography, Department for Earth System Sciences, Universität Hamburg, Bundesstraße 55, 20146 Hamburg, Germany
[3]Helmholtz-Zentrum hereon GmbH, Department of Human Dimensions in Coastal Areas, Max-Planck-Straße 1, 21502 Geesthacht, Germany
[4]E2S UPPA, Chair HPC-Waves, SIAME, Université de Pau et des Pays de l'Adour, Allée du parc Montaury, Anglet, France
[5]University of Hawai'i at Mānoa, Department of Oceanography, 1000 Pope Road, Honolulu, HI 96822, USA
[6]formerly Maldives Meteorological Service, Hulhule' 22000, Maldives
[*]david@lufi.uni-hannover.de

1 Photoscan Settings

Photoscan is performing a three-dimensional, virtual reconstruction of the coastal areas of Fuvahmulah, based on the aerial images, recorded in three field campaigns. Tab. 1 shows the settings within the software for each reconstruction. The settings resulted in digital elevation models (DEMs) with a similar resolution of $\approx 3.5\,\mathrm{cm\,px^{-1}}$.

Table 1. Photoscan settings to obtain digital elevation models from the coastal areas, recorded in each field campaign.

	**2017 March**<br>Dry Season	**2017 September**<br>Wet Season	**2019 March**<br>Dry Season
**Alignment**			
Accuracy	High	Medium	High
Generic preselection	Yes	Yes	Yes
Reference preselection	Yes	Yes	Yes
Key point limit	40000	40000	40000
Tie point limit	4000	4000	4000
Adaptive camera model fitting	No	Yes	Yes
Optimization Parameters	f, cx, cy, k1-k4 (k1-k4, p1-p4)[1]	f, b1, b2, cx, cy, k1-k4, p1-p4	f, cx, cy, k1-k4
**Dense Point Cloud**			
Quality	Ultra High	Medium	High
Depth filtering	Aggressive	Aggressive	Aggressive
**Mesh**			
Surface type	Height field	Height field	Height field
Source data	Dense Point Cloud	Dense Point Cloud	Dense Point Cloud
Quality	Ultra High	Medium	High
Depth filtering	Aggressive	Aggressive	Aggressive
**DEM**			
Source data	Mesh	Dense cloud	Mesh
**Software**			
Agisoft Photoscan Version	1.4.5 build 7354	1.4.5 build 7354	1.4.5 build 7354
Platform	Linux (Ubuntu) 64	Linux (Ubuntu) 64	Linux (Ubuntu) 64

[1] for the adjacent 350 m north of the harbour.

2 Wave Statistics

Tables 2 and 3 display the associated median and IQR values for the boxplots of the significant wave height H_s around Fuvahmulah, as seen in Fig. 5 of the main manuscript. The data in Table 2 is based on the Collaboration for Australian Weather and Climate Research (CAWCR) wave hindcast collection[1] for the time period 1979 – 2019. Table 3 contains statistical values for the Representative Concentration Pathway (RCP) projections 4.5 and 8.5 of the CAWCR wave projections[2] for the time frame 2081 – 2100 and the associated historical H_s values for the period 1986 – 2005. The methods of the main article contain further information on the wave data collection.

Table 2. Statistic values of the significant wave height H_s for the CAWCR wave hindcast data set (1979 – 2019), as shown in Fig. 5a of the main article

Month	Median H_s (m)	IQR of H_s (m)	Month	Median H_s (m)	IQR of H_s (m)
January	1.04	0.21	July	1.71	0.33
February	0.99	0.24	August	1.64	0.31
March	0.98	0.23	September	1.48	0.31
April	1.15	0.33	October	1.37	0.40
May	1.38	0.35	November	1.23	0.39
June	1.62	0.34	December	1.07	0.29

Table 3. Statistic values of the significant wave height H_s for the historical (1986 – 2005) and RCP data set (2081 – 2100), as shown in Fig. 5f of the main article

RCP	Median H_s (m)	IQR of H_s (m)
Historical	1.62	0.60
RCP 4.5	1.63	0.61
RCP 8.5	1.62	0.61

3 Current Velocities

Since the study focuses on the wave driven longshore currents, ocean circulation around Fuvahmulah and the tidal current velocity was disregarded for sediment transport processes. To justify this simplification, the following section compares velocities from ocean circulation, tidal current velocities and wave-induced currents with each other. The velocity of the ocean circulation was derived from monthly means of the European Centre for Medium-Range Weather Forecasts (ECMWF) Ocean ReAnalysis System 5 (ORAS5) reanalysis data-set[3]. This reanalysis data set calculates ocean (thermo-) dynamics and provides zonal and meridional velocities between 1979 – 2017 at the position 0.0° latitude and 73.4° longitude (see Fig. 1 of the main article). According to the ORAS5 data, the median ocean circulation velocity In this region is $v_{m,\text{oceancirc.}} = 0.40\,\text{m}\,\text{s}^{-1}$. The tidal current velocities are calculated by Delft3D (D3D). The simulation uses the University of Hawai‘i Sea-Level Center (UHSLC) time-series[4] of the neighboring Gan tide gauge ($\sim 53\,\text{km}$ away from Fuvahmulah). The D3D computations gave a median tidal current velocity of $v_{m,\text{tide.}} = 0.10\,\text{m}\,\text{s}^{-1}$ with two peaks on the northwest ($v_{max,\text{tide.}} = 0.30\,\text{m}\,\text{s}^{-1}$) and southeast-side ($v_{SE,\text{tide.}} = 0.28\,\text{m}\,\text{s}^{-1}$) of the island. These velocities are an order of magnitude below wave-induced currents (Fig. 7 of the main article) and thus can be neglected. This can be considered standard practice when regarding longshore sediment transport and confirms that longshore sediment transport mainly depends on incident waves, while tidal and ocean currents are of minor importance[5].

4 Selected Questions of the Questionnaires

This section presents relevant questions from the population survey and their answer possibilities (closed questions) or most frequent answers (open questions), as well as associated codes and coding rules:

Question 1: The following list shows environmental issues concerning the coast. In your opinion, which 3 are the most pressing issues for Fuvahmulah?
(Closed-ended question; 3 responses possible per respondent, Respondents=116; Responses=345)

- Waste (22 %)
- Coral Bleaching (4 %)
- Declining fish populations (4 %)
- Flooding from rainfall (7 %)
- Coral mining (2 %)
- Too ambitious fishing regulations (1 %)
- Declining rainfall (6 %)
- Erosion (27 %)
- Loss of land (7 %)
- Tsunami (1 %)
- Over-fishing (1 %)
- Sand mining (15 %)
- Coastal flooding (1 %)
- Other (5 %)

Question 2: Have you observed any changes regarding the natural environment of Fuvahmulah in the past? If yes, please tell me what changes you have observed and since when.
(Hybrid question, Respondents=116)

- Yes (93 %)
  (Open-ended, multiple responses possible per respondent; Respondents=108, Responses=218)
  Three most often named categories:
  Codes (Coding rule, Example statement):
  - Erosion (36 %)
    Statement names erosion as an environmental change.
    Example: *"Due to high currents and huge waves – the soil from coastal areas are wiped out."* (S1 063)
  - Less trees (15 %)
    Statement states that less trees are found on the island as an environmental change.
    Example *"Trees are less."* (S1 111)
  - Temperature increase (7 %)
    Statement names increase of temperature as an environmental change.
    Example *"It's getting hotter."* (S1 084)
- No (7 %)

Question 3: Do you feel safe to live in Fuvahmulah with regard to sea-level rise?
(Open-ended question, categorized, Respondents=116)

Codes (Coding rule, Example statement):

- Feels safe (52 %)
  Statement demonstrating that the respondent feels safe on the island without doubt.
  Example: *"Currently the safest island in Maldives is Fuvahmulah because it has highest coast."* (S1 026)
- Does not feel safe (25 %)
  Statement demonstrating that the respondent does not feel safe on the island.
  Example: *"No, it's not safe. I would prefer to move to some other island."* (S1 076)
- Inconclusive (22 %)
  Statement does not allow a clear valuation.

Example: *"If we get flood than we won't survive. This island's coasts are higher but middle of island is lower. It's like a bowl."* (S1 073)

- Don't know (1 %)

Question 4: What are elements of the natural environment of Fuvahmulah that are important to you?
(Open-ended question, categorized, multiple responses possible per person; Respondents=116; Responses=235)

Codes (Coding rule, Example statement):

- Trees (17 %)
  Statement mentions trees as an important element of the natural environment.
  Example: " *"Trees around the island."* (S1 114)
- Beaches (8 %)
  Statement mentions the beach as an important element of the natural environment.
  Example: " *"Nice beach."* (S1 049)
- Lakes (8 %)
  Statement mentions lakes as an important element of the natural environment.
  Example: " *"North lake near the house. It's very peaceful and in the evening, we go there with our children."* (S1 012)

Question 5: How is the community involved in the decision-making process regarding the development of the island?
(Open-ended question, categorized, Respondents=115)

Codes (Coding rule, Example statement):

- Regular involvement (17 %)
  Statement arguing that community is included in the decision-making process.
  Example: *"Are included. Good things and bad things come out of it."* (S1 031)
- Sporadic involvement (17 %)
  Statement arguing that the community is included in the decision-making process from time to time or in limited ways.
  Example: *"Sometimes they take our opinions, and sometimes they just go ahead with it."* (S1 033)
- Insufficient involvement (49 %)
  Statement clearly demonstrating dissatisfaction with how the community is involved in the decision-making process.
  Example: *"Not involved. The leaders of the community decide it for themselves."* (S1 107)
- Inconclusive (7 %)
  Statement does not allow a clear valuation.
  Example: *"Don't know about other, but if I am asked for involvement, I go to help them."* (S1 109)
- Don't know (8 %)

Question 6: Would you want to be more involved in the decision-making processes regarding the development of the island?
(Closed-ended question, Respondents=116)

- Yes (83 %)
- No (15 %)
- Don't know (2 %)

Supplementary Information for the article:
Considering socio-political framings when analyzing coastal climate change effects can prevent maldevelopment on small islands (David et al., 2021)

Data availability

The field data[6] from the aerial surveys and bathymetry for numerical modeling is publicly available through doi.org/10.5281/zenodo.4304049.
Wave climate data is publicly available from the respective services and homepages of CAWCR (CSIRO Data Acess Portal), ECMWF (Copernicus Climate Data Store) and National Oceanic and Atmospheric Administration (NOAA) (WAVEWATCH III®Hindcast and Reanalysis Archives). For further information of the climate data, see also Table 1 in the main article.
Source data supporting the social sciences' finding are provided within the supplementary materials of this paper. Other raw data (for example interview transcripts) on the social sciences part that support the findings of this study are protected and not publicly available as they contain personal information that could compromise research participant privacy. Other data from the social science part can be made available from the social science team (B.M.W.R. and A.H.) upon reasonable request.

Code availability

The aerial images were processed with the Structure-from-Motion MultiView Stereo (SfM-MVS) algorithm of Agisoft Photscan Pro (version 1.4.5., build 7354). Except for the bathymetry of the numerical model, geo-data was processed with Quantum GIS 3.10 A Coruña and 3.16 Hannover on Ubuntu Linux 18.04 and 20.04 and/or Python modules rasterio v.1.1.1. to v.1.1.3. together with the module Fiona v.1.8.13.
The Boussinesq Ocean and Surf Zone (BOSZ, version 01-2019) model is available through Volker Roeber upon reasonable request. The Generic Mapping Tool (GMT, version 5.4.5) was used to process the geo-data to be used as bathymetry in BOSZ. Other simulations on the morphodynamics around the reef island was carried out with Delft3D. Delft3D is open-source and available through Deltares (oss.deltares.nl/web/delft3d). In the Delft 3D Suite, we used the modules Deltares FLOWD3D 6.02.13.9162M, SWAN III 40.72ABCDE.
Other scripts for data post processing and visualization, are written with the open-source software Python 3.7 in Jupyter Notebooks, using the modules Numpy 1.17.4, Matplotlib (pyplot) 3.1.2, Xarray 0.12.1 with NetCDF 1.5.3, Pandas 0.25.3, cartopy 0.18.0 and cdsapi 0.1.3 and later versions. Cartopy uses OpenStreetMap data (available under the Open Database License, see openstreetmap.org) as well as Stamen open source maps.
Data of the second household survey and interviews were analyzed with the software MAXQDA.

Supplementary Information for the article:
Considering socio-political framings when analyzing coastal climate change effects can prevent maldevelopment on small islands (David et al., 2021)

References

1. Durrant, T., Hemer, M., Smith, G., Trenham, C. & Greenslade, D. CAWCR Wave Hindcast - Aggregated Collection. v1 (2019). Last accessed: 2020-03-29, permalink: hdl.handle.net/102.100.100/137152?index=1.

2. Hemer, M., Trenham, C., Durrant, T. & Greenslade, D. CAWCR Global wind-wave 21st century climate projections. v1 (2015). Last accessed: 2020-03-29, DOI: doi.org/10.4225/08/55C991CC3F0E8.

3. Zuo, H., Balmaseda, M. A., Tietsche, S., Mogensen, K. & Mayer, M. The ECMWF operational ensemble reanalysis–analysis system for ocean and sea ice: a description of the system and assessment. *Ocean. Sci.* **15**, 779–808, DOI: 10.5194/os-15-779-2019 (2019).

4. Caldwell, P. C., Merrifield, M. A. & Thompson, P. R. Sea level measured by tide gauges from global oceans — the Joint Archive for Sea Level holdings (NCEI Accession 0019568). Dataset. Version 5.5, NOAA National Centers for Environmental Information, (2015). DOI: 10.7289/V5V40S7W.

5. Burcharth, H. F., Hawkins, S. J., Zanuttigh, B. & Lamberti, A. Design tools related to engineering. In Burcharth, H. F., Hawkins, S. J., Zanuttigh, B. & Lamberti, A. (eds.) *Environmental Design Guidelines for Low Crested Coastal Structures*, 203 – 333, DOI: doi.org/10.1016/B978-008044951-7/50033-6 (Elsevier Science Ltd, Oxford, 2007).

6. David, C. G., Ballesteros, P. & Schlurmann, T. Coastal Digital Elevation Models and Transects of the Reef Island Fuvahmulah, the Maldives [Dataset]. *Zenodo* DOI: 10.5281/zenodo.4304049 (2020).

Acknowledgments

This study took place in the project "Dealing with change in SIDS: societal action and political reaction in sea level change adaptation in Small Island Developing States (DICES)", grant no. SCHL 503/17-1 (CGD, TS) and RA 585/19-1 (AH, BR). The project is framed within the priority programm (SPP-1889) - regional sea level change and society of the German Research Foundation (Deutsche Forschungsgemeinschaft, DFG).

Volker Roeber acknowledges financial support from the Isite program Energy Environment Solutions (E2S), the Communauté d'Agglomération Pays Basque (CAPB) and the Communauté Région Nouvelle Aquitaine (CRNA) for the chair position HPC-Waves; as well as the support from the University of Hawai'i at Mānoa for the Affiliate Graduate Faculty position.

Zahid was employed as Deputy Director General Climatology at the Maldives Meteorological Service (MMS) at the time of the project, but retired from his position.

The authors would like to thank Ali Ahmed, Pablo Ballesteros, Tatiana Ivanova, René Klein, Nina Kohl, Manò Schütt, Ibrahim Shiyan (Panda), Jailam Zahir as well as Marion and Uwe Zander for their help in the field campaigns. In the Maldives, the authors were supported by the Maldives Meteorological Service (MMS), Fuvahmulah Island Council and Fuvahmulah DIVE School. Pablo Ballesteros and René Klein are staff of Ludwig-Franzius-Institute, Leibniz University of Hanover (LuFI-LUH) and supported the field campaign with their technical support. Nina Kohl and Manò Schütt were student assistants at LuFI, helping to record data on Fuvahmulah. Ali Ahmed and Jailam Zahir were locals and assisted the in the coastal surveys (Ali) and in the interviews and household survey (Jailam).

The authors also thank Tilo Schöne of German Research Centre for Geosciences, Helmholtz Centre Potsdam (GFZ) Potsdam, providing the satellite radar altimetry (SRA) data as well as Jean Bidlot (ECMWF), Mark Hemer (Australian Commonwealth Scientific and Industrial Research Organisation, CSIRO) and Todd Spindler (National Centers for Environmental Protection (NCEP)-Environmental Modeling Center (EMC)) for their help in accessing climate reanalysis data. Also, Elisa Casella and Alessio Rovere gave valuable insights and feedback to unmanned aerial vehicle (UAV)-based photogrammetry, while Tobias Kersten helped with geodetic questions before the third field campaign and with post-processing the global navigation satellite system (GNSS) data. Furthermore, the authors acknowledge Jannek Gundlach for his feedback on and Astrid Kartes and Jonas Briese for their assistance with Delft3D. Both, Astrid and Jonas, were students at LuFI during the project.

Supplementary Information for the article:
Considering socio-political framings when analyzing coastal climate change effects can prevent maldevelopment on small islands (David et al., 2021)

Author Contribution

C.G.D. conducted the natural science / engineering part of field campaigns, research and analysis, while A.H. carried out the corresponding social science part. V.R. developed the BOSZ model used in this study and supervised the numerical modeling. T.S. and B.R. (co-)designed the research project, were responsible for funding resources and reporting and provided guidance throughout the entire research. C.G.D. wrote the manuscript with input from A.H., while B.R., V.R., T.S. and Z. edited and contributed to the final manuscript. Z. helped in designing the research project, coordinating the field surveys and provided a local perspective to the evaluation and analysis.

Conflict of Interest Statement

The authors declare no competing interests.

Ethics Statement

All participants gave their informed consent and we complied with all relevant ethical regulations. Ethical affairs have been handled and declared during the funding phase of the project. Ethics committee and institutional review board approval was determined not to be required for this project.

Acronyms

2DH	depth-integrated
ADS	Altimeter Data System
C3S	Copernicus Climate Change Service
CAWCR	Collaboration for Australian Weather and Climate Research
CFSR	Climate Forecast System Reanalysis
CFSv2	Climate Forecast System Reanalysis v.2
CMIP5	Coupled Model Intercomparison Project Phase 5
CSIRO	Australian Commonwealth Scientific and Industrial Research Organisation
D3D	Delft3D
DEM	digital elevation model
DPSIR	drivers, pressures, state, impact and response
ECMWF	European Centre for Medium-Range Weather Forecasts
EIA	Environmental Impact Assessment
EMC	Environmental Modeling Center
ERA5	fifth generation atmospheric reanalysis of the global climate
GCP	ground control point
GFZ	German Research Centre for Geosciences, Helmholtz Centre Potsdam
GNSS	global navigation satellite system
IFS	Integrated Forecasting System
IPCC	Intergovernmental Panel on Climate Change
IQR	interquartile range
MSL	mean sea level
NCEP	National Centers for Environmental Protection
NOAA	National Oceanic and Atmospheric Administration

Supplementary Information for the article:
Considering socio-political framings when analyzing coastal climate change effects can prevent maldevelopment on small islands (David et al., 2021)

RCP	Representative Concentration Pathway
SfM	structure from motion
SRA	satellite radar altimetry
SROCC	Special Report Ocean and Cryosphere in a Changing Climate
UAV	unmanned aerial vehicle
vGCP	virtual Ground Control Point
WAM	ocean wave model
WGS84	World Geodetic System
