# Supplementary material for: Considering socio-political framings when analyzing coastal climate change effects can prevent maldevelopment on small islands
Source: Nat Commun. 2021 Oct 7;12:5882. doi: 10.1038/s41467-021-26082-5 (PMC8497557; doi:10.1038/s41467-021-26082-5)
Supplement: Supplementary file 3 — Reporting Summary [file 41467_2021_26082_MOESM3_ESM.pdf]

nature research

C. Gabriel David, M.Sc.

Leibniz Universität Hannover
Ludwig-Franzius-Institute for Hydraulic,
Estuarine and Coastal Engineering
Nienburger Str. 4
30167 Hannover - Germany

Corresponding author(s): eMail: david@lufi.uni-hannover.de

Last updated by author(s): Jul 22, 2021

Reporting Summary

Nature Research wishes to improve the reproducibility of the work that we publish. This form provides structure for consistency and transparency in reporting. For further information on Nature Research policies, see our Editorial Policies and the Editorial Policy Checklist.

Statistics

For all statistical analyses, confirm that the following items are present in the figure legend, table legend, main text, or Methods section.

n/a	Confirmed	
☒	☐	The exact sample size (*n*) for each experimental group/condition, given as a discrete number and unit of measurement
☒	☐	A statement on whether measurements were taken from distinct samples or whether the same sample was measured repeatedly
☒	☐	The statistical test(s) used AND whether they are one- or two-sided<br>*Only common tests should be described solely by name; describe more complex techniques in the Methods section.*
☒	☐	A description of all covariates tested
☒	☐	A description of any assumptions or corrections, such as tests of normality and adjustment for multiple comparisons
☐	☒	A full description of the statistical parameters including central tendency (e.g. means) or other basic estimates (e.g. regression coefficient) AND variation (e.g. standard deviation) or associated estimates of uncertainty (e.g. confidence intervals)
☒	☐	For null hypothesis testing, the test statistic (e.g. *F*, *t*, *r*) with confidence intervals, effect sizes, degrees of freedom and *P* value noted<br>*Give P values as exact values whenever suitable.*
☒	☐	For Bayesian analysis, information on the choice of priors and Markov chain Monte Carlo settings
☒	☐	For hierarchical and complex designs, identification of the appropriate level for tests and full reporting of outcomes
☒	☐	Estimates of effect sizes (e.g. Cohen's *d*, Pearson's *r*), indicating how they were calculated

Our web collection on statistics for biologists contains articles on many of the points above.

Software and code

Policy information about availability of computer code

Data collection

Data requests (collection) from the global reanalysis / wave hindcast models was carried out with Python version 3.7 with modules Numpy 1.17.4, Xarray 0.12.1 with NetCDF 1.5.3 as well as cdsapi 0.1.3 and later versions.

Data analysis

Data post processing:
- General data post processing was done with Python 3.7 and modules Numpy 1.17.4, Xarray 0.12.1 with NetCDF 1.5.3, Matplotlib (pyplot) 3.1.2, Pandas 0.25.3 and cartopy 0.18.0.

Geoinformation Data:
- Structure-from-Motion MultiView Stereo (SfM-MVS) procedure: Agisoft Photoscan Pro (version 1.4.5, build 7354)
- GIS Software: Generic Mapping Tool (Command line version 5.4.5 stable) for bathymetry in the numerical model, for all other geo-data Quantum GIS 3.10 A Coruña LTR and 3.16 Hannover on Ubuntu Linux 18.04 and 20.04* and/or Python modules rasterio v.1.1.1 to v.1.1.3* together with Fiona v.1.8.13.

Numerical Modelling:
- Delft 3D Suite using Deltares FLOWD3D 6.02.13.9162M, SWAN III 40.72ABCDE
- Boussinesq Ocean and Surf Zone model (BOSZ - see Roeber, 2010**; Roeber and Cheung, 2012***) version 01-2019

The authors are happy to provide Python scripts in form of Jupyter Notebooks upon reasonable request.

Until made publicly available, the BOSZ code is available through Volker Roeber, also upon reasonable request.

* software was updated during review process. ** doi.org/10.1016/j.coastaleng.2009.11.007, *** doi.org/10.1016/j.coastaleng.2012.06.001

For manuscripts utilizing custom algorithms or software that are central to the research but not yet described in published literature, software must be made available to editors and reviewers. We strongly encourage code deposition in a community repository (e.g. GitHub). See the Nature Research guidelines for submitting code & software for further information.

Data

Policy information about availability of data

All manuscripts must include a data availability statement. This statement should provide the following information, where applicable:

- Accession codes, unique identifiers, or web links for publicly available datasets
- A list of figures that have associated raw data
- A description of any restrictions on data availability

Data availability:
The field data supporting the findings of this study as well as the bathymetry for BOSZ is publicly available through doi.org/10.5281/zenodo.4304049.
Wave climate data is available from the respective services and homepages of CAWCR, ECMWF and NOAA (links in the main manuscript, Table 1 as well as Data Availability section).

Field-specific reporting

Please select the one below that is the best fit for your research. If you are not sure, read the appropriate sections before making your selection.

☐ Life sciences ☐ Behavioural & social sciences ☒ Ecological, evolutionary & environmental sciences

For a reference copy of the document with all sections, see nature.com/documents/nr-reporting-summary-flat.pdf

Ecological, evolutionary & environmental sciences study design

All studies must disclose on these points even when the disclosure is negative.

Study description

--- Coastal engineering / Natural & environmental sciences: ---
Three field campaigns were conducted to gather topographic and bathymetric data for the numerical models and to evaluate the seasonal and annual erosion processes around the island: The first campaign started in March 2017 at the end of the dry season. The second field campaign took place at the end of the wet season in September 2017. The last field measurements were taken in March and April 2019. Each field campaign had a duration of about one month (see section field "Timing and spatial scale").
The first field campaign accommodated a bathymetric survey, recording depth profiles with a dual-frequency echo-sounder (Dr. Fahrentholz LituBox 15/200) on the entire Fuvahmulah reef. The bathymetry was then used in the numerical models.
Furthermore, each campaign has a systematically recorded collection of UAV-borne images for the SfM-MVS procedure and photogrammetric reconstruction of the nearshore reef and beaches of Fuvahmulah. To bring the topographic models into a global coordinate system, GNSS position measurements were carried out (Trimble 4700 and 5700 receiver and Septentrio AsteRx system).
The photogrammetric reconstruction resulted in topographic information of the beaches and the reef, which was then also used in the numerical models and to estimate erosion on Fuvahmulah's coast.
Wave climate information was taken provided by CAWCR, ECMWF and NOAA-EMC reanalysis or wave hindcast databases (links in the main document)

--- Social sciences ---
- Population survey:
The study applied a triangulation of different empirical methods, including expert interviews, population survey, participatory observation and mapping. The central part of this mixed-method approach was a structured random population study in the case study area. The explorative approach started with a series of expert interviews in the nation's capital Malé and with the atoll administration of Fuvahmulah. This allowed us to acquire official statements and documents, as well as diverse opinions and attitudes towards an under-researched topic in this part of the world. The semi-structured questionnaire of survey 1 included structured as well as open-ended questions. Survey 2 encompassed only open-ended questions. All ethical policies and procedures were closely adhered to for this minimal risk study. All responses were voluntary, confidential, and anonymous, data was safeguarded and further anonymized

- Interviews:
Purposive sampling. The selection of interview partners was based interviewee's profession and expertise on the subject.

Research sample

--- Coastal engineering / Natural & environmental sciences ---
Wave and topographic data was recorded in the dry and subsequent wet season, to analyze seasonal changes and between dry season in 2017 and dry season in 2019 to analyze interannual changes.

- Wave data.
The study compares wave data from three data sources (provided by CAWCR, ECWMF and NCEP; see main article) and Satellite Radar Altimetry (SRA) measurement. The study uses hourly (CAWCR, ECMWF) or 3-hourly (NCEP) wave data between 1980-01-01 until 2019-05-31 in the cross-correlation function when comparing the different data sets among each other (n=345504 when comparing CAWCR with ECMWF; n=115168 when comparing NCEP with CAWCR and ECMWF data, the latter two were downsampled to the equivalent 3-hourly data). The SRA measurement for Fuvahmulah contains 337 data points.
For all further wave data analysis, this study refers to the hourly data from the CAWCR data set between 1980 to 2019 (40 years

including end date = 14 610 days = 350640 data points).
The evaluation of future climate change impact is done according to the IPCC's 5th assessment report baseline with 6-hourly hindcast data between 1986-2005 (n=204467, NaN's are left out) and equivalent 6-hourly wave data in the time period 2081 – 2100 (end of the century) under RCP 4.5 and RCP 8.5 projections (both n=230203, NaN's are left out).

- Bathymetry:
Bathymetry was recorded using a dual-frequency echo-sounder (Dr. Fahrentholz LituBox 15/200, sampling rate 5Hz) together with a GNSS antenna and a processing unit (Raspberry Pi 2) in the field campaign in 2017. The echo-sounder was mounted on a fishing boat, traveling multiple times around the island (see section field "Timing and spatial scale").

- Digital elevation models (DEMs):
DEMs are reconstructed in Photoscan with the following data samples:
2017, dry season: 1.316 aerial images, 182 markers, split up in 4 chunks.
2019, dry season: 2.533 aerial images, 420 markers, split up in 4 chunks.

--- Social sciences ---
- Population survey:
All participating inhabitants of the island were above the age of 14.

Survey 1:
116 participants;
62 female, 54 male;
Age groups: 14-19yrs: 12, 20-25yrs: 13, 26-31yrs: 19, 32-37yrs: 25, 38-43yrs: 12, 44-49yrs: 10, 50-55yrs: 5, 56-61yrs: 8, 62-67yrs: 6, 68-73yrs: 4, >73: 2

Survey 2:
98 participants;
51 female, 47 male;
Age groups: 14-19yrs: 7, 20-25yrs: 6, 26-31yrs: 16, 32-37yrs: 23, 38-43yrs: 13, 44-49yrs: 6, 50-55yrs: 12, 56-61yrs: 8, 62-67yrs: 8, 68-73yrs: 5, >73: 4
The study was representative in regard of age and gender. The sample's sex ratio are similar distributed than the resident population of Fuvahmulah, according to the 2014 Census.

- Interviews:
Actors on national level:
10 government, 8 non-governmental or intergovernmental organizations and researchers;
Actors on local level:
8 government, 6 non-governmental organizations

Sampling strategy

--- Coastal engineering / Natural & environmental sciences ---
- Bathymetry:
The strategy was systematic, subsequent sampling of the reef around the island in multiple sessions. The southern reef was recorded in one session (Images of the boat track is available upon reasonable request from the author). The sampling strategy facilitated recording sufficient bathymetry data, with a much higher resolution and better accuracy than publicly available sources. The bathymetry resolution is high enough to study wave propagation and transformation over the reef in the numerical model.

- Aerial images:
In 2017, systematic, low-altitude flights of each survey site were carried out and low-tide, as well as low-altitude flights at the end of the campaign where they were possible. The 2019 field campaign focused mainly on low-tide, low-altitude images, as these provided the best quality in the post processing before. In all campaigns, the entire coastline was recorded, except the beach in front of the airport (this recordings contain of 9.3km of a total 11km beach length).
The 420 markers in the field campaign 2019 were Ground Control Points (GCPs), measured with survey-grade GNSS equipment. The GNSS measurements on the beaches were corrected by a constant reference measurement (Javad Legacy GNSS receiver, with GNSS antenna mounted on a roof), collecting data throughout the entire 2019 field campaign. The 2017 field campaign had no correction by a constant reference measurement. To improve the accuracy within the WGS84, virtual Ground Control Points (vGCPS) of specific landmarks, available in both models, georeferenced the 2017 DEMs with the 2019 DEMs.
With that, very high resolution topographic data was recorded, allowing to reveal seasonal and inter-annual coastal erosion in sub-decimeter scale.

--- Social sciences ---
A systematic sampling strategy was applied, addressing one member of every eighth household with our interviews, giving a representative subsample of Fuvahmulah's inhabitants. The number of participants was determined by the duration and thus available time during the field campaigns. If nobody was at the selected house, the nearest neighboring house was chosen, and the selection continued from there on to maintain the target subsample size (and thus validity and significance of the survey).
Interviews were conducted from Saturday to Thursday (Maldivian weekdays), beginning at 9am and stopping at 7pm. This allowed us to contact all population groups. In respect to the religious practices, Friday was not used for the survey.

Data collection

--- Coastal engineering / Natural & environmental sciences ---
Assistants: Ali Ahmed, Pablo Ballesteros, René Klein, Nina Kohl, Manó Schütt
- Bathymetry:
One set of water depth, recorded with a dual-frequency echo-sounder with a sampling rate of 5Hz. We hired two fisher, steering the boat and a local to translate.

- Aerial images:
Aerial images were recorded with a DJI Phantom 4 (2017) and a DJI Phantom 4 Pro (2019). Both devices are Unmanned Aerial

Vehicles (UAVs). Flight planning was done with DroneDeploy and DJI GroundStation.
The GCPs were recorded with GNSS equipment (Trimble 4700 and 5700 receiver, Zephyr and 13" Trimble GPS antennas (2017) and 2 Septentrio AsteRx-U receiver in base and rover mode with NAVX-3G antennas (2019). The constant reference measurements were carried out with a Javad Legacy GNSS receiver (base station), connected to a NAVX-3G antenna, mounted on a roof. The Septentrio and Javad receivers produced raw observation data to correct the rover positions via RTKLIB 2.4.3 b31.

The beach profiles of adjacent airport beaches were recorded with the aforementioned GNSS equipment.
All UAV flights and beach profile measurements were carried out in accordance and under the acknowledgement of the local airport administration. Local airport staff also assisted with equipment transport on the airport properties.

--- Social sciences ---
Assistant: Jailam Zahir
- Population survey:
The following instruments were used to collect the data: pen and paper (both surveys), photos/illustrations of alternative coastal protection measures (survey 1) and an audio recorder (survey 2). However, 70% of the interviewees did not want to be recorded. In order to guarantee a high level of data quality, the research assistant was trained in the techniques of proper scientific conduction of surveys beforehand.

- Interviews:
Following instruments were used to collect the data: audio recorder, pen and paper. Interviews were conducted in English when possible, otherwise local research assistants conducted the interviews in the local language Dhivehi. A research assistant was also present in the interviews conducted by the researcher. Using a local research assistant was recommended by Maldivian research institutions, because people would be unwilling to engage if foreigners were to ask for and conduct an interview without a local assistant.
The researcher was not blind to the study hypothesis during data collection.

Timing and spatial scale

--- Coastal engineering / Natural & environmental sciences ---
- Field Campaigns:
1: March 2nd, 2017 - March 23rd, 2017
2: August 25th, 2017 - September 30th, 2017
3: March 13th, 2019 - April 7th, 2019
Spatial Scale: the island and fringing reef of Fuvahmulah (approx. position: latitude -0.275° to -0.335°; longitude 73.41° to 73.45°).

- Bathymetry:
Bathymetry was recorded in 7 surveys within 5 days (morning sessions from dawn to noon; afternoon sessions from afternoon to dusk).

- SfM-MVS procedure:
Aerial images captured the entire coast of Fuvahmulah, except the beaches adjacent to the airport area (approximately from the southern end of the waste dump to the west side of the seaport). Spatially, each UAV survey was limited by the GNSS system's radio range, covering about 100-150m of the beach. Time wise, each UAV survey took 15-25mins for GCP recording, 15mins flight time and 10-15mins. GCP marker collection. The entire data set contains recordings of 9.3km of a total 11km beach length.

- Other elevation data:
GNSS survey of the airport beaches required two sessions (one day, morning and afternoon).

--- Social sciences ---
Field trip 1: 7.3.2017 – 13.4.2017; Survey 2: 14.3.2017 – 6.4.2017
Field trip 2: 14.01.2019 – 22.02.2019; Survey 2: 21.1.2019 – 14.2.2019
Spatial scale: the island of Fuvahmulah (approx. position: latitude -0.30°; longitude 73.43°) and the capital Malé (approx. position: latitude 4.17°; longitude 73.45°)

Data exclusions

--- Coastal engineering / Natural & environmental sciences ---
No SfM-MVS data for the beaches adjacent to the airport. The area is supplemented with beach profiles from manual measurements. In terms of coastal topography, the study only required data for the east coast of Fuvahmulah, other data was not shown here (but in David and Schlurmann, 2020)*.

--- Social sciences ---
No data was excluded
Non-participation: Approximately 25% of the inquired persons in both surveys declined interviews. Reasons given were time constraints or no interest.

*doi.org/10.3389/fmars.2020.538675

Reproducibility

--- Coastal engineering / Natural & environmental sciences ---
Except for the bathymetry, each of the three field campaigns contained measurements of the same coastal areas. Apart from the erosion, the position of dedicated landmarks were reconstructed with expectable accuracy when using survey-grade GNSS equipment and SfM-MVS procedures (for an overview on expectable accuracy, see Table 1 in Casella et al., 2020*).

--- Social sciences ---
There were no experiments undertaken in the study site. The population survey information is provided under items "data collection" and "randomization".

*doi.org/10.1007/s00367-020-00638-8

Randomization

--- Coastal engineering / Natural & environmental sciences ---
The study deals with measured and data and data from numerical models. Therefore, no randomization is applicable.

--- Social sciences ---
The sample size of each survey is equivalent to about every eighth household on the island, while household selection was done by previous randomized sampling, yielding an unbiased representation of local households.

Blinding

--- Coastal engineering / Natural & environmental sciences ---
The study deals with measured and data and data from numerical models. Therefore, no blinding is applicable.

--- Social sciences ---
A randomization rule was applied during the selection of the households (see section "randomization") blinding is not possible in this context. However, as a basic condition of their participation, we guaranteed the respondents unrestricted anonymity and abstraction of their statements in the evaluation of the social science study.

Did the study involve field work? ☒ Yes ☐ No

Field work, collection and transport

Field conditions

The dry season featured high temperatures around 30 C°, had no to small amounts of rainfall and low wave heights. The wet or rainy season was subject to similar temperatures with almost daily, sudden and heavy rainfalls and high(er) waves. The circumstances, weather and climate were typical for the Maldives.

Location

Latitude approx. between -0.275° and -0.335°
Longitude approx. between 73.41° and 73.45°

Access & import/export

Access via Int. Airport Velana in Malé and then by the local airport on Fuvahmulah.
Customs were handled with a logistics company in 2017 and in 2019 by the researchers themselves. The project was supported by the Maldives Meterological service (co-author Dr. Zahid), who also handled official allowances to conduct the field campaigns as well as any custom related issues. Approval of the survey was issued by the Maldives' National Bureau of Statistics through the "Request for Survey Approval" document in advance of the field campaign. The approval form was a prerequisite for our entry permit with customs declaration on the day of entry at Male airport.
UAV flights on the island were in general legal, but the researchers additionally consulted with the local airport administration, who approved the surveys. Further approval of the field campaign was given by the local island administration (town hall and major).

Disturbance

No disturbances came up or were caused.

Reporting for specific materials, systems and methods

We require information from authors about some types of materials, experimental systems and methods used in many studies. Here, indicate whether each material, system or method listed is relevant to your study. If you are not sure if a list item applies to your research, read the appropriate section before selecting a response.

Materials & experimental systems

n/a	Involved in the study	
☒	☐	Antibodies
☒	☐	Eukaryotic cell lines
☒	☐	Palaeontology and archaeology
☒	☐	Animals and other organisms
☐	☒	Human research participants
☒	☐	Clinical data
☒	☐	Dual use research of concern

Methods

n/a	Involved in the study	
☒	☐	ChIP-seq
☒	☐	Flow cytometry
☒	☐	MRI-based neuroimaging

Human research participants

Policy information about studies involving human research participants

Population characteristics

see "Ecological, evolutionary & environmental sciences study design", field "Research sample", section "Social sciences".

Recruitment

The expert interviews in the nation's capital and with the atoll's administration were based on the interviewee's role in coastal protection and climate change adaptation. A systematic sampling strategy was applied for the population survey, addressing one member of every eighth household with our interviews. The number of participants was determined by the time available within each field campaign. If nobody was at available at a selected house, the nearest neighboring house was chosen, and the survey continued from there on.
Interviews were conducted by the researcher in English when possible, otherwise the local research conducted in the local language Dhivehi. In order to guarantee a high level of data quality, the research assistant was trained in the techniques of

proper scientific conduction of surveys beforehand. Using the help of a local research assistant was recommended by Maldivian research institutions, because people would be unwilling to engage if foreigners were to ask for and conduct an interview without a local assistant.
Within the framework of the measures taken to ensure ethically sound, transparent and structured data collection, we see no risk of potential self-selection bias. The balance between reapprochement and distance to the study participants was maintained at all times. The evaluative analysis took place cooperatively and in reflective loops together with our counterparts in the Maldives.

Ethics oversight

All ethical policies and procedures were closely adhered to for this minimal risk study. All responses were voluntary, confidential, and anonymous; data was safeguarded and further anonymized.

Note that full information on the approval of the study protocol must also be provided in the manuscript.
